# RELEAP: reinforcement-enhanced label-efficient active phenotyping for electronic health records

**DOI:** 10.1093/jamiaopen/ooag019

**Published:** 2026-02-18

**Authors:** Yang Yang, Kathryn I Pollak, Bibhas Chakraborty, Molei Liu, Doudou Zhou, Chuan Hong

**Affiliations:** Department of Biostatistics and Bioinformatics, Duke University, Durham, NC 27705, United States; Cancer Prevention and Control Research Program, Duke Cancer Institute, Durham, NC 27710, United States; Department of Population Health Sciences, Duke University School of Medicine, Durham, NC 27701, United States; Department of Biostatistics and Bioinformatics, Duke University, Durham, NC 27705, United States; Department of Biostatistics, Peking University Health Science Center, Beijing 100191, China; Beijing International Center for Mathematical Research, Peking University, Beijing 100871, China; Department of Statistics and Data Science, National University of Singapore, 117546, Singapore; Department of Biostatistics and Bioinformatics, Duke University, Durham, NC 27705, United States

**Keywords:** reinforcement learning, active learning, phenotyping, risk prediction, lung cancer

## Abstract

**Objectives:**

Electronic health record (EHR) phenotyping often relies on noisy proxy labels, which undermine the reliability of downstream risk prediction. Active learning can reduce annotation costs, but typical heuristics do not directly optimize downstream prediction. Our goal was to develop a framework that directly uses downstream prediction performance as feedback to guide phenotype correction and sample selection under constrained labeling budgets.

**Materials and Methods:**

We propose reinforcement-enhanced label-efficient active phenotyping (RELEAP), a reinforcement learning-based active learning framework. Reinforcement-enhanced label-efficient adaptively integrates multiple querying strategies and, unlike prior methods, updates its policy based on feedback from downstream models. We evaluated RELEAP on a de-identified Duke University Health System (DUHS) cohort (2014-2024) for incident lung cancer risk prediction, using logistic regression and penalized Cox survival models. Performance was benchmarked against noisy-label baselines and single-strategy active learning.

**Results:**

Reinforcement-enhanced label-efficient improved over the proxy-only baseline and approached oracle performance under the same budget. Logistic AUC increased from 0.774 to 0.807. Survival concordance index increased from 0.715 to 0.749. Gains were stable across iterations using downstream feedback. These trends were consistent in sex-stratified subgroup analyses (female vs male).

**Discussion:**

By linking phenotype refinement to prediction outcomes, RELEAP learns which samples most improve downstream discrimination and calibration, offering a more principled alternative to fixed active learning rules.

**Conclusion:**

Reinforcement-enhanced label-efficient optimizes phenotype correction through downstream feedback, offering a scalable, label-efficient paradigm that reduces manual chart review and enhances the reliability of EHR-based risk prediction.

## Introduction

Phenotyping refers to the process of deriving clinically meaningful traits, such as diseases, behaviors, or risk factors, from raw health data using structured codes, laboratory results, medications, and unstructured clinical text. Intermediate phenotypes play a central role in biomedical prediction models, serving as covariates or mediators that connect underlying patient characteristics to clinical outcomes. Their accuracy directly shapes the performance, interpretability, and fairness of downstream risk prediction. Within electronic health records (EHRs), however, such phenotypes are rarely observed directly. Instead, they must be inferred from raw data sources, including diagnoses, procedures, laboratory results, medications, and clinical text, using computable phenotypes, algorithmically defined constructs based on EHR data (eg, diagnosis codes, medications, laboratory values, or unstructured clinical text) that approximate clinical conditions or behaviors.^1–3^

Developing high-quality computable phenotypes at scale remains a major bottleneck. While unsupervised approaches like clustering and rule-based heuristics avoid the need for labels, they often lack specificity and clinical validity.^4^^,^^5^ Supervised learning offers higher accuracy but depends on manually curated labels derived from chart review, which is time-intensive, costly, and unscalable. These challenges have sparked growing interest in label-efficient phenotyping strategies, which aim to reduce annotation burden without compromising phenotype quality. Approaches such as weak supervision,^6^ surrogate labeling,^7^ and active learning exemplify this shift toward minimizing reliance on gold-standard labels.^8^

The accuracy of label-efficient phenotyping can be significantly enhanced by leveraging multiple data modalities. Structured EHR elements, such as International Classification of Diseases (ICD) codes,^9^ are often used as proxies for behavioral phenotypes like smoking, but their sensitivity is low and subject to substantial label noise.^10^ For instance, studies have shown that relying solely on structured codes captures only a small subset of true smokers and systematically underrepresents smoking prevalence.^11^ Extracting information from unstructured clinical text, ranging from traditional natural language processing (NLP) to recent large language models (LLMs), provides a more complete phenotype than structured codes alone.^12^^,^^13^ In this study, we specifically leverage LLMs to extract high-fidelity smoking mentions to construct our automatic reference phenotype,^14^ thereby reducing misclassification and forming a robust foundation for label-efficient learning.

A further complication is that while small validation cohorts from chart review can be used to benchmark algorithms, such labels are insufficient for monitoring performance across diverse populations or for guiding iterative refinement. This limitation motivates the use of downstream prediction performance to guide active learning rather than relying exclusively on scarce gold-standard labels. A related line of work has sought to directly connect phenotyping with risk prediction. For example, Hong et al. proposed a semi-supervised framework that jointly validates multiple surrogate phenotypes while estimating associations with genetic risk factors.^15^ By combining the phenotyping step with the downstream risk genetic association study, their method improved both phenotype accuracy and the reliability of subsequent genetic association analyses. This illustrates the value of integrating phenotype refinement with predictive modeling, though existing approaches have largely focused on semi-supervised inference rather than adaptive sample selection. Conceptually, our framework aligns with two-phase study designs and multi-wave validation sampling in biostatistics^16–18^ where an expensive gold standard is obtained for a subsample to correct measurement errors in the full cohort. However, while traditional designs primarily focus on unbiased parameter inference, RELEAP shifts the objective toward downstream predictive performance, utilizing a feedback-driven policy to optimize discrimination metrics under a fixed budget.

To facilitate consistent terminology, we define the automatic reference phenotype as a high-fidelity label that integrates structured self-reported smoking information with LLM-based extraction from clinical notes. This multimodal phenotype serves as the most accurate available reference for downstream evaluation and is denoted as $S_{\text{true}}$ throughout this paper. Importantly, although structured self-reported smoking fields are available in our Duke University Health System (DUHS) data, we intentionally define the proxy phenotype $S^{*}$ using only smoking-related ICD codes. This design choice mirrors common EHR settings where self-reported smoking variables are sparse, inconsistently captured across sites, or entirely unavailable (eg, certain ICU datasets or claims-based sources). In RELEAP, $S^{*}$ represents the readily available but noisy structured proxy, whereas $S_{\text{true}}$ represents a higher-fidelity reference phenotype that integrates stronger signals (eg, self-report and note-based evidence) and is treated as a higher-cost (computationally or operationally) reference label that is not assumed to be routinely available at scale, so we simulate budget-constrained access to $S_{\text{true}}$. This separation yields a conservative evaluation: it tests whether the reinforcement learning-active learning (RL-AL) mechanism can efficiently close the gap from a weak, widely available proxy ($S^{*}$) toward a higher-fidelity reference phenotype ($S_{\text{true}}$) when only a limited number of reference labels can be accessed. This choice reflects a portability-oriented design rather than a judgment about data quality, as self-reported smoking variables are not consistently available across EHR systems.

Building on this definition, we present reinforcement-enhanced label-efficient active phenotyping (RELEAP), a reinforcement learning-driven active learning agent designed to improve phenotype correction under constrained labeling budgets.^19^^,^^20^ Unlike prior label-efficient approaches, RELEAP directly uses downstream risk prediction performance as a feedback signal for phenotype refinement. By leveraging automatically constructed reference labels instead of manual chart review, RELEAP is both scalable and cost-efficient. Building on prior work such as Hong et al.,^15^ we benchmark RELEAP against noisy-label and single-strategy baselines and quantify label efficiency by the labeling budget required to approach near-oracle performance. In the remainder of the paper, we describe the RELEAP framework and experimental setup, then report results for both logistic and survival prediction tasks. We additionally present sex-stratified (female vs male) subgroup analyses to illustrate how querying strategies may behave differently across population distributions.

## Materials and methods

### Motivation example: lung cancer prediction with imprecise smoking phenotypes

Lung cancer remains a leading cause of cancer-related mortality, and risk prediction models are increasingly being developed to support earlier detection and preventive care. Recent machine learning models trained on large-scale EHR data have demonstrated strong performance in identifying high-risk patients, achieving area under the receiver operating characteristic curve (AUC) values above 0.75 across diverse real-world settings.^21^ In these models, smoking behavior consistently emerges as one of the most important predictors, alongside age, race, and chronic respiratory conditions such as chronic obstructive pulmonary disease (COPD).^22^

However, accurately capturing smoking status in EHR data is challenging. In routine care, smoking behavior may be incompletely recorded and updated over time. Diagnosis codes related to tobacco use are therefore often used as computable proxies, but they can be sparse and insensitive for behavioral exposures. In our analysis of EHR data from the DUHS, we found that the ICD-based proxy smoking phenotype ($S^{*}$) is markedly incomplete when compared to self-reported smoking status. While structured self-report fields in the vital signs table indicate that approximately 30% of patients have a documented history of smoking, only about 10% of patients carry any smoking-related ICD-9/10 code (eg, 305.1, F17.*, Z87.891) prior to the index date. This discrepancy highlights the limitations of relying on diagnosis codes alone and suggests substantial under-ascertainment when ICD codes are used as a proxy phenotype.

While self-reported smoking status can be a strong structured signal when available, we do not use it to define $S^{*}$ in the main experiments for 2 primary reasons. First, structured self-report fields are not consistently available or comparable across different health systems and datasets, whereas diagnosis codes are more consistently available and standardized in both EHR and claims data, supporting the portability of our framework to settings with limited structured elements. Second, RELEAP is designed to evaluate how a limited labeling budget can be strategically allocated to upgrade a widely available but noisy proxy label ($S^{*}$) toward a higher-fidelity reference label ($S_{\text{true}}$). Using ICD codes to construct $S^{*}$ therefore creates a realistic and stringent setting in which label noise and sparsity are present by design, allowing for a rigorous evaluation of the active learning mechanism.

To address this gap, we constructed an automatic reference phenotype by combining structured self-reported smoking information with unstructured smoking mentions extracted from clinical notes using LLMs prior to the prediction index date. This multimodal definition provides a more complete and accurate depiction of patient smoking history and serves as a reference label for evaluation. The discrepancy between $S^{*}$ and $S_{\text{true}}$ highlights a budget-constrained decision problem: given limited access to high-fidelity reference labels, determining which patients should be upgraded from noisy proxies in order to maximize downstream prediction performance. This decision problem motivates the design of RELEAP, which adaptively allocates scarce label queries rather than relying on fixed heuristics.

### Proposed RELEAP agent

**Figure 1.**
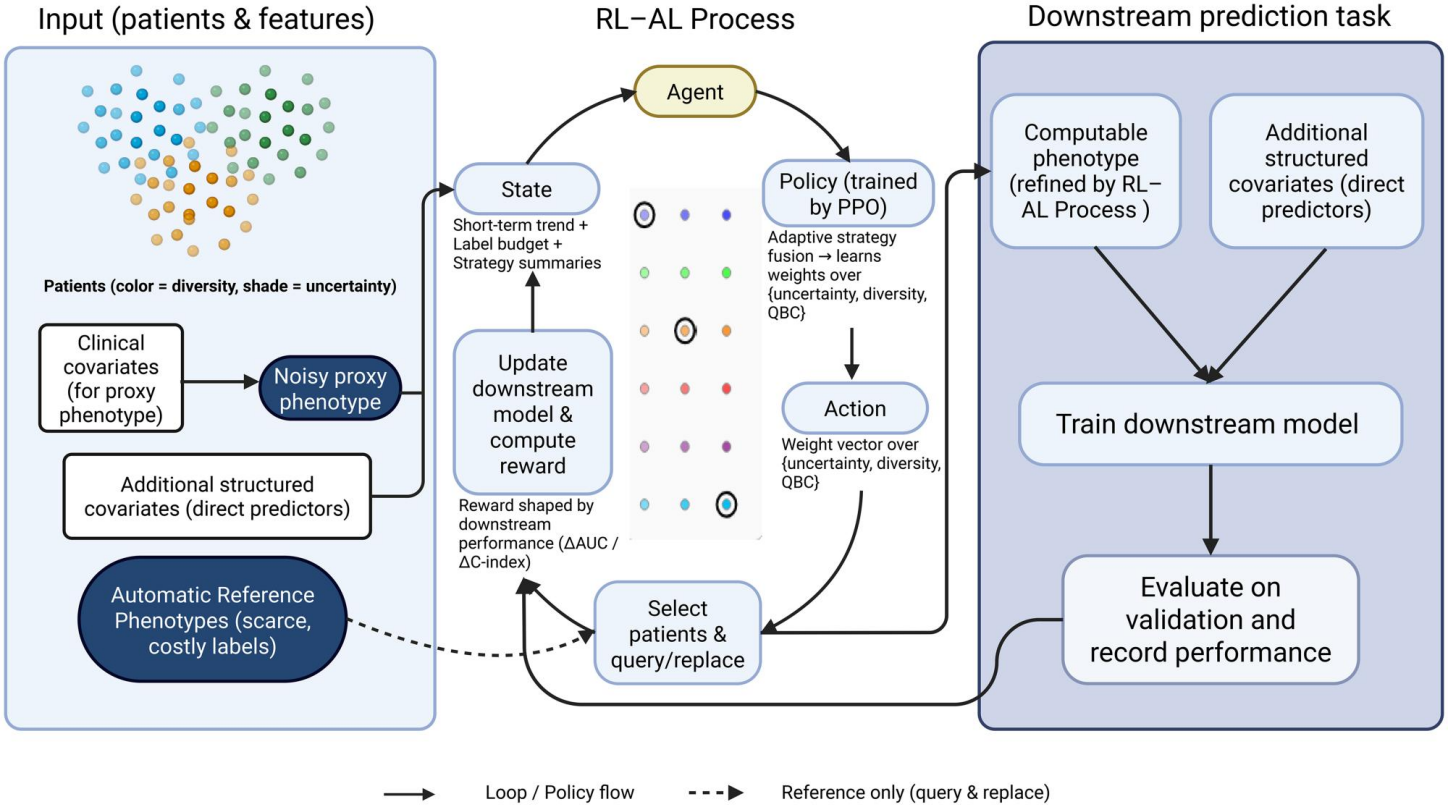
RELEAP workflow. Patient-level inputs include clinical covariates for constructing proxy phenotypes, additional structured predictors for risk models, and a limited pool of costly reference labels. The reinforcement learning-active learning (RL-AL) process adaptively selects patients to correct noisy proxy phenotypes into refined computable phenotypes, guided by a policy trained with proximal policy optimization (PPO). The state vector summarizes short-term downstream performance trends, labeling budget, and strategy-level statistics. The policy outputs weights across active learning strategies (uncertainty, diversity, and query-by-committee, QBC), which drive the action of selecting patients for relabeling. Corrected phenotypes are then combined with additional structured predictors to train downstream risk prediction models. Model performance on validation data (eg, area under the receiver operating characteristic curve [AUC] or concordance index [C-index]) is logged as the final outcome and also fed back as the reward signal, closing the loop between downstream prediction and the RL-AL process.

The RELEAP workflow (Figure 1) starts from a proxy phenotype $S^{*}$ constructed from structured inputs $X_{1}$ and a small seed set with reference labels $S_{\text{true}}$. In our application, $S_{\text{true}}$ is the automatic reference phenotype integrating structured self-reported smoking status and LLM-extracted note evidence prior to the index date. A downstream lung cancer risk model provides performance feedback throughout training (binary outcome *Y*; time-to-event *T* in survival analyses). At each iteration, candidate unlabeled patients are scored using multiple active learning heuristics (uncertainty, diversity, and query-by-committee, QBC), as described in the “Active Learning Strategies” section. An RL agent then adaptively learns how to weight these strategies to select the most informative samples, with detailed reward formulations provided in Supplementary Section S3.

Throughout training, RELEAP maintains a phenotype label vector $S_{t}$ over the training set. We initialize $S_{0}=S^{*}$ and iteratively update $S_{t}$ by replacing proxy values with $S_{\text{true}}$ for queried patients. In our experiments, the automatic reference phenotype $S_{\text{true}}$ is constructed for the full cohort, but it is only revealed to the learner for queried patients to emulate budget-constrained access to high-fidelity labels. The downstream model is retrained after every iteration, and a shaped reward based on predictive performance is used to update the RL policy. Training episodes terminate when the labeling budget is exhausted. This closed feedback loop allows RELEAP to balance exploration and exploitation,^19^ progressively aligning corrected phenotypes with true patient characteristics and improving predictive accuracy and calibration.

#### Active learning strategies

We consider 3 active learning strategies (uncertainty, diversity, and QBC) as the action basis for the RELEAP agent. At each iteration, candidate patients are scored by these strategies, normalized, and then combined by the agent to select the top-*k* samples. Random sampling is included as a comparator but is not part of the RL action space.

All features are standardized as *z*-scores computed on the current labeled training set. For labeled patients, we use the automatic reference phenotype label $S_{\text{true}}$, whereas for unlabeled patients we rely on the continuous proxy score $S^{*}$. To ensure comparability, all strategy scores are min–max normalized to the range [0,1] in each iteration. Ties are resolved by adding a small random jitter ($\epsilon \approx 10^{-6}$). Once selected, patients are labeled without replacement and are never re-queried.

The strategies capture complementary notions of informativeness:

1. *Uncertainty*. This strategy prioritizes patients whose outcomes are most ambiguous under the current downstream model. For each unlabeled patient *i*, uncertainty is quantified by the entropy of the predicted probability ${\hat{p}}_{t,i}$:
  $$H({\hat{p}}_{t,i})=-{\hat{p}}_{t,i}\;\text{log}\;{\hat{p}}_{t,i}-(1-{\hat{p}}_{t,i})\;\text{log}(1-{\hat{p}}_{t,i}),$$
  favoring samples for which the model is least confident.^23^
2. *Diversity*. While uncertainty targets ambiguous cases, diversity promotes coverage across the feature space. Distances are computed between each unlabeled patient and its nearest labeled neighbors, with higher scores assigned to patients farther from the labeled set. A variance adjustment is applied for numerical stability (see Supplementary Section S2).^24^
3. *Query-by-Committee (QBC)*. To capture model disagreement, we train a committee of M=7 logistic regression models on bootstrap resamples of the labeled set. Each model applies mild perturbations, including small random jitter in the L2 penalty and feature dropout with probability 0.1. The QBC score reflects disagreement across committee models, measured by the prediction variance with an entropy-based stabilizer (see Supplementary Section S2). This strategy favors patients where competing models disagree most.^25^

#### Reinforcement learning integration

We model phenotype correction under a label budget as a partially observable Markov decision process (POMDP).

*Notation*. We index active learning iterations by t=1,…,H, where *H* is the number of iterations (planning horizon) until the labeling budget is exhausted. At iteration *t*, the agent observes a summary state $s_{t}$, selects an action $a_{t}$, receives a scalar reward $R_{t}$, and transitions to the next state $s_{t+1}$. Strictly, in a POMDP the agent receives observations $o_{t}$; here, we use $s_{t}=\phi (h_{t})$, a constructed summary derived from the interaction history $h_{t}$, as the state input to the policy. We denote the stochastic policy by $\pi_{\theta }(a|s)$ with parameters θ, which specifies a distribution over actions given by the current state. In RELEAP, the action corresponds to a strategy-mixture (weight) vector $w_{t}=(w_{t}^{\text{unc}},w_{t}^{\text{div}},w_{t}^{\text{qbc}})\in \Delta^{2}$, and we set $a_{t}\equiv w_{t}$ (defined below). The policy is trained to maximize the expected discounted return $E[\sum_{t=1}^{H}\gamma^{t-1}R_{t}]$, where γ∈(0,1] is the discount factor that controls the emphasis on long-term vs short-term gains.

*State*. The state $s_{t}$ summarizes the current learning context, including per-strategy score summaries on the unlabeled pool, composition of the labeled set, the recent trajectory of downstream performance, and the remaining budget (see Supplementary Section S3 for full details).

*Action*. The action at iteration *t* is a weight vector over the 3 strategies,


$$a_{t}\equiv w_{t}=(w_{t}^{\text{unc}},w_{t}^{\text{div}},w_{t}^{\text{qbc}}),\;w_{t}^{i}\geq 0\;\;(i\in \{\text{unc},\text{div},\text{qbc}\}),\;\sum_{i\in \{\text{unc},\text{div},\text{qbc}\}}w_{t}^{i}=1.$$

Thus, $a_{t}$ lies on the probability simplex $\Delta^{2}$ and defines how per-sample strategy scores are combined into a single ranking for sample selection. In implementation, the policy network outputs unconstrained logits, which are transformed by a softmax function to produce valid weights that are nonnegative and sum to 1. Note that the softmax is applied to the policy logits (mixture coefficients), not to the raw per-sample strategy scores (eg entropy) themselves.

*Reward*. The agent receives a shaped reward $R_{t}$ computed from downstream validation performance after the phenotype update at iteration *t* (eg, AUC in classification or concordance index [C-index] in survival). Concretely, $R_{t}$ reflects the improvement in the validation metric from iteration t−1 to *t*, with additional stabilization using a short moving baseline and mild budget-aware scaling (full details in Supplementary Section S3).

*RL algorithm*. We optimize the policy $\pi_{\theta }(a|s)$ using proximal policy optimization (PPO) to maximize the expected discounted return $E[\sum_{t=1}^{H}\gamma^{t-1}R_{t}]$, where *H* denotes the episode length (budget exhaustion).

*Iteration loop*. Each iteration follows an agent–environment cycle: the agent observes $s_{t}$, selects an action $a_{t}=w_{t}$, receives reward $R_{t}$, and transitions to $s_{t+1}$. The practical implementation proceeds as follows:

1. Compute strategy scores on the unlabeled pool using uncertainty, diversity, and QBC.
2. Observe the current state $s_{t}$, including strategy summaries, recent trajectory, budget fraction, labeled-set composition, and downstream performance.
3. Select an action $w_{t}$ via the PPO policy and rank the unlabeled pool accordingly.
4. Acquire labels by querying $S_{\text{true}}$ for the top-*k* selected patients and update the phenotype label vector by overwriting proxy values for queried patients. Specifically, we initialize $S_{0}=S^{*}$ and define $S_{t}$ as the updated label vector after iteration *t*, where queried patients use $S_{\text{true}}$ and all remaining patients continue to contribute through their $S^{*}$ values.
5. Retrain the downstream prediction model (logistic or penalized Cox, depending on model) and evaluate validation performance; full details on feature processing and additional metrics are in Supplementary Section S3.
6. Compute the reward $R_{t}$, store $\left(s_{t},a_{t},R_{t},s_{t+1}\right)$, and update the PPO agent periodically.

Details of the simulation setup and synthetic data experiments are provided in Supplementary Section S1.

*Simulation summary*. In synthetic experiments under the minimal structure $X_{1}\to S_{\text{true}}\to S^{*}$ and $(S_{\text{true}},X_{2})\to Y$, all active learning strategies improved over the proxy-only baseline and moved toward the oracle trained on fully observed $S_{\text{true}}$. RELEAP was generally competitive with strong fixed heuristics (eg, uncertainty sampling and QBC), with relative differences depending on the iteration and data setting. When the outcome was rare (1%), strategy differences were more pronounced and performance gains accrued more gradually, making sample selection more consequential. As prevalence increased (10% and 30%), all methods converged more quickly, and the between-strategy gaps narrowed. Details are provided in Supplementary Section S1.

### Evaluation of RELEAP on real-world EHR data

We evaluated RELEAP using de-identified EHR data from the DUHS spanning 2014-2024. This dataset provides a representative testbed for assessing phenotype correction in the context of lung cancer risk prediction, where accurate characterization of smoking behavior is particularly critical. As noted earlier, ICD-only proxies ($S^{*}$) tend to underreport smoking compared to the multimodal automatic reference phenotype ($S_{\text{true}}$), motivating evaluation in this setting.

#### Cohort and study population

The study cohort included adults aged 35-70 years with at least 365 days of prior EHR history before the index date, defined as the qualifying outpatient encounter. Duplicate patient records were removed. Patients with evidence of prevalent lung cancer prior to the index date were excluded. Records with missing demographic information (eg, sex, race) were retained; missing categorical values were encoded with an explicit “Unknown” category and missing continuous values were imputed using the training-set median (details in the “Preprocessing” section). After applying these criteria, the final cohort included *N* = 238 119 patients.

#### Variable construction and notation

Study variables were derived from both structured and unstructured EHR elements. Smoking-related ICD-9/10 codes were extracted within a 1-year look-back window prior to the index date and encoded as features ($X_{1}$). A proxy phenotype ($S^{*}$) was defined as the predicted probability from an ICD-only logistic regression model: we trained a logistic regression using $X_{1}$ to predict a binary smoking label derived from structured self-reported smoking status (current/former vs never) when available, and then applied the fitted model to all patients to obtain $S^{*}$. No additional covariates were incorporated in this proxy model. The automatic reference phenotype ($S_{\text{true}}$) was defined by combining structured self-reported smoking status with LLM-extracted smoking mentions from clinical notes prior to index, prioritizing structured entries in case of disagreement. Additional structured covariates ($X_{2}$) included demographics, COPD (ICD-9/10-based binary indicator and 1-year preindex count), and a Hispanic ethnicity indicator, all measured at baseline; $X_{2}$ was used exclusively in the downstream outcome models and excluded the smoking-related ICD codes in $X_{1}$. The outcome (*Y*) was incident lung cancer after the index date, defined as the presence of ≥2 primary lung cancer diagnosis codes (ICD-9: 162.0–162.9; ICD-10: C34.0–C34.9, including all subcodes) recorded within 60 days of each other before the administrative study end date. Time-to-event (*T*) was measured from the index date to the date of the first lung cancer diagnosis code in the qualifying pair; patients without an event were administratively censored at the study end date (complete code lists are provided in Supplementary Section S6).

#### Automatic reference phenotype definition and benchmark variants

To provide additional context requested by reviewers, we compared multiple automatic reference phenotype definitions that incorporate (1) structured self-reported smoking information and (2) smoking signals extracted from clinical notes using either traditional NLP or LLMs. Unless otherwise noted, our primary automatic reference phenotype $S_{\text{true}}$ integrates structured self-reported smoking status with smoking-related mentions extracted from clinical notes using an LLM.

As benchmark variants, we additionally evaluated: (1) SR + NLP CUIs: structured self-report combined with smoking-related concept unique identifiers (CUIs) extracted by traditional NLP, (2) NLP CUIs-only (no SR): CUIs extracted by traditional NLP without self-reported information, (3) LLM mentions-only (no SR): LLM-extracted smoking mentions without self-reported information and (4) SR-only: structured self-reported smoking status without note-derived signals. Comparative results for these benchmark variants are summarized in Supplementary Section S5.

#### Preprocessing

**Figure 2.**
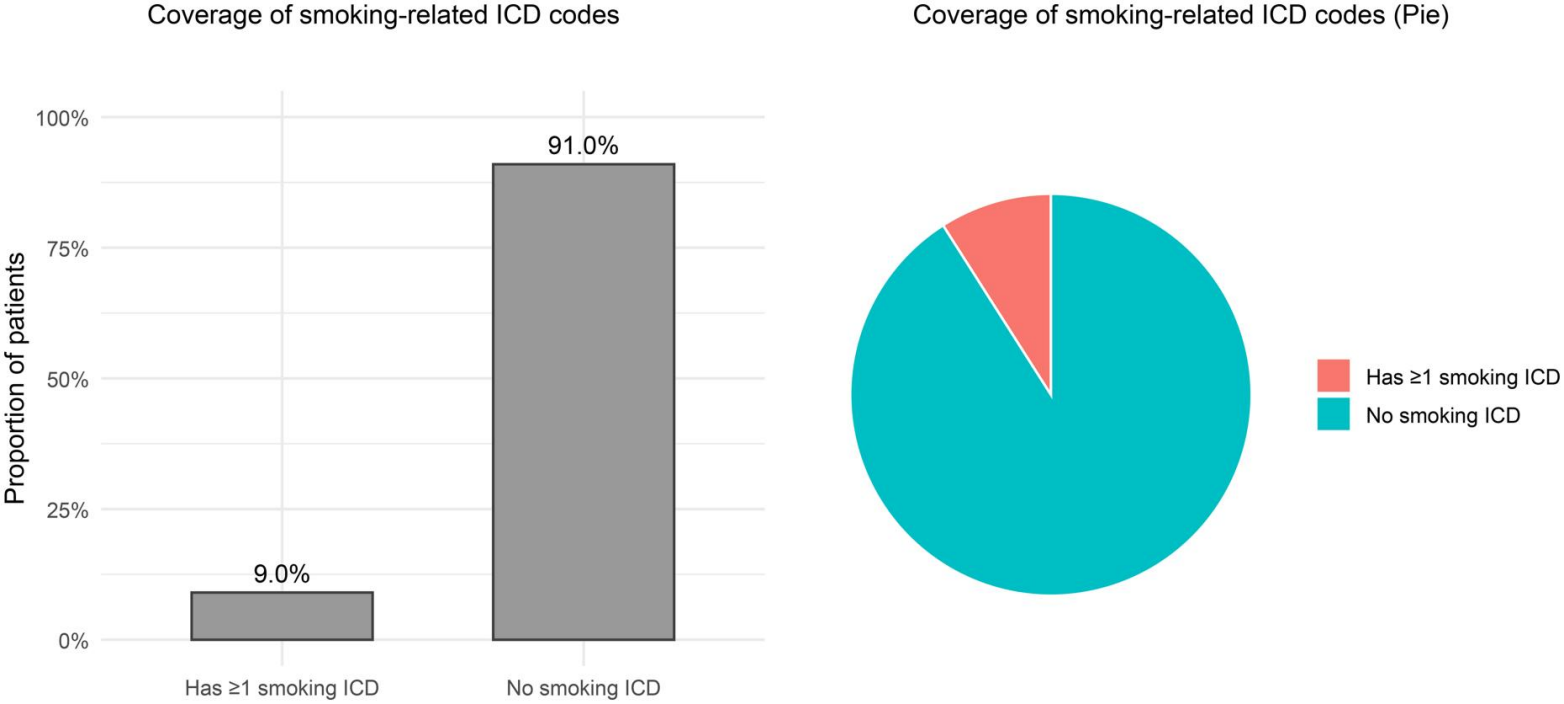
Coverage of smoking-related ICD codes (presence of ≥1 smoking-related ICD per patient). Abbreviation: ICD, International Classification of Diseases.

Categorical variables were one-hot encoded with an explicit “Unknown” category for missing values. Continuous variables were standardized, and missing values were imputed using the training-set median. All features and $S_{\text{true}}$ were constructed strictly from pre-index data to prevent data leakage. A descriptive summary of the final cohort, including demographic characteristics, smoking phenotype distributions (based on $S_{\text{true}}$), outcome rates, COPD prevalence, and coverage of smoking-related ICD codes, is provided in Table 1. Figure 2 further illustrates the proportion of patients with at least 1 smoking-related ICD code.

**Table 1.** Dataset summary of the DUHS lung cancer cohort.

Characteristic	Value
Overall	
*N*	238 119
Age, mean (SD)	52.94 (10.23)
Age, median (IQR)	53 (44-62)
Sex	
Female	138 415 (58.1%)
Male	99 702 (41.9%)
Unknown	2 (0.0%)
Race	
American Indian or Alaska Native	1512 (0.6%)
Asian	8357 (3.5%)
Black or African American	57 014 (23.9%)
Multiple race	926 (0.4%)
Native Hawaiian or other Pacific Islander	227 (0.1%)
No information	318 (0.1%)
Other	4184 (1.8%)
Refuse to answer	6227 (2.6%)
White	159 354 (66.9%)
Hispanic/Latino	
Declined to report	8844 (3.7%)
No	219 828 (92.3%)
Unavailable	187 (0.1%)
Unknown	24 (0.0%)
Yes	9236 (3.9%)
Smoking	
Never smoked	125 556 (52.7%)
No information	14 940 (6.3%)
Smoked in the past	50 301 (21.1%)
Smokes daily	19 717 (8.3%)
Smokes daily (heavy)	204 (0.1%)
Smokes daily (light)	1345 (0.6%)
Smokes some days	3863 (1.6%)
Smoking status unknown	89 (0.0%)
Unknown	22 104 (9.3%)
Outcome	
Lung cancer, *n* (%)	1846 (0.78%)
Smoking ICD coverage	
No smoking ICD	216 614 (91.0%)
Has ≥1 smoking ICD	21 505 (9.0%)
COPD	
No	226 055 (94.9%)
Yes	12 064 (5.1%)

*SEX* values are expanded to full names; *RACE* uses single-code mapping (01-07, NI, UN, OT); *HISPANIC* is reported separately (Y, N, R, UN, NI). *Smoking behavior* was derived from vitals labels in column smoking_status and presented as behavioral descriptors (Smokes daily/Smokes some days/Smoked in the past/Never smoked/Unknown). *Heavy* and *light* correspond to vitals labels (eg, “heavy tobacco smoker” and “light tobacco smoker”); no quantitative cigarettes-per-day definition was available in this dataset. *Smoking ICD coverage* is defined as having ≥1 smoking-related ICD codes.

Abbreviations: COPD, chronic obstructive pulmonary disease; DUHS, Duke University Health System; ICD, International Classification of Diseases.

### Baseline and comparator methods

We compare RELEAP against commonly used non-adaptive baselines, including a weakly supervised noisy-label approach (Aphrodite) and fixed-strategy active learning heuristics (uncertainty, diversity, and QBC used alone without RL weighting). We also include random sampling as a reference baseline.

#### Aphrodite-based baseline

As a pragmatic comparator, we implemented the Aphrodite method,^26^ a noisy-label baseline that relies on structured ICD codes and NLP-derived CUIs without adaptive querying. Positive examples were seeded by the presence of smoking-related ICD codes (eg, V15.82, 305.1, F17.*, Z72.0, Z87.891, Z71.6) and/or affirmed NLP CUI mentions, whereas negative examples required no smoking-related ICD codes, no affirmed CUI mentions, and zero pre-index notes. Using these seeds, we trained a regularized logistic regression model on per-code counts (ICD/CUI) augmented with a note-count feature, and then scored all patients. This noisy but scalable baseline follows the Aphrodite framework for weakly supervised phenotype labeling in EHR data.

#### Random and fixed-strategy baselines

In addition to Aphrodite, we evaluate random sampling and fixed-strategy active learning baselines that query patients using uncertainty, diversity, or QBC alone (ie, without RL weighting). These baselines use the same iteration budget, feature processing, and downstream evaluation as RELEAP to enable fair comparisons.

*Comparators*. We benchmarked RELEAP against the following comparator methods: (1) *Proxy-only* ($S^{*}$ baseline): a zero-label baseline trained directly on the noisy proxy phenotype ($S^{*}$) without label correction, (2) *Aphrodite*: a weakly supervised method relying on structured ICD codes and NLP-derived CUIs without adaptive querying, (3) *Random sampling*: active learning with random querying, (4) *Uncertainty sampling*: querying by uncertainty alone, (5) *Diversity sampling*: querying by diversity alone, (6) *QBC*: querying by QBC alone, (7) *RELEAP*: a performance-driven fusion of multiple sampling strategies via RL, and (8) *Oracle* ($S_{\text{true}}$ oracle): a fully supervised upper bound trained with all high-fidelity automatic reference phenotype labels ($S_{\text{true}}$). For completeness, we also compared RELEAP across multiple automatic reference phenotype definitions described in the “Evaluation of RELEAP on Real-World EHR Data” section and summarized results in Supplementary Section S5.

### Experimental setup

Experiments followed a training-validation framework to simulate real-world phenotype correction. Patients were randomly split into 80% training and 20% validation subsets, stratified by the outcome to preserve event rates. Active learning began with a small, balanced seed set drawn from the training partition, where the noisy proxy labels ($S^{*}$) were replaced by the automatic reference labels ($S_{\text{true}}$) to initialize the labeled set. All remaining training patients continued to contribute through their proxy labels, ensuring that the entire training pool was used for model updates. At each iteration, the RELEAP agent generated a set of weights across candidate sampling strategies to guide patient selection. For evaluation, the same weighting scheme was mirrored on the validation set so that training and validation evolved under comparable replacement dynamics. Downstream prediction models were retrained at every iteration, and their predictive performance was tracked in parallel. In our primary experiments, the active learning budget queries a total of 82 000 reference labels from the training set per replication (approximately 43.1% of the training pool; 34.4% of the full cohort *N* = 238 119). We therefore report labeling budgets both as absolute counts and as proportions.

### Planned analyses

The primary classification task predicted incident lung cancer using the combined feature set of the current phenotype label vector ($S_{t}$) and structured covariates ($X_{2}$). Model performance was primarily evaluated by the AUC. A secondary analysis used a penalized Cox proportional hazards model to estimate time-to-event outcomes, with predictive accuracy summarized by the C-index. In this survival model, we define *Z* as the covariate vector formed by the current corrected phenotype value together with additional structured predictors after feature screening, that is, $Z=(S_{t},X_{2})$. The Cox model is written as $h(t|Z)=h_{0}(t)\;\text{exp}(\beta^{⊤}Z)$. Performance across iterations was summarized as the mean value with 95% CIs across repeated simulations. Final-round comparisons included measures of discrimination (AUC or C-index), calibration (mean squared error [MSE] of predicted probabilities), and threshold-based metrics such as the F1 score, true positive rate (TPR), and positive predictive value (PPV) at a fixed false positive rate of 0.1. We also conducted subgroup analyses stratified by sex, not for clinical inference but to illustrate how different active learning strategies can behave differently under varying data distributions.

## Results

We evaluated RELEAP on the DUHS lung cancer cohort to assess its effectiveness in correcting noisy proxy phenotypes. Results are organized into 2 parts: (1) overall performance across all patients, considering both logistic classification and survival analyses and (2) subgroup analyses stratified by sex to illustrate how different active learning strategies behave under varying data distributions.

### Overall performance across all patients

RELEAP was evaluated under 2 settings, logistic classification and survival analysis. In both models, RELEAP substantially improved predictive performance over the noisy proxy phenotype baseline ($S^{*}$) and approached the oracle trained with all automatic reference phenotype labels ($S_{\text{true}}$). Benchmark comparisons (Supplementary Section S5) showed that SR+LLM and SR+NLP definitions achieved the strongest discrimination and calibration, while text-only variants (no SR) were consistently weaker and SR-only provided an intermediate baseline.

#### Logistic classification model (evaluated by AUC)

**Figure 3.**
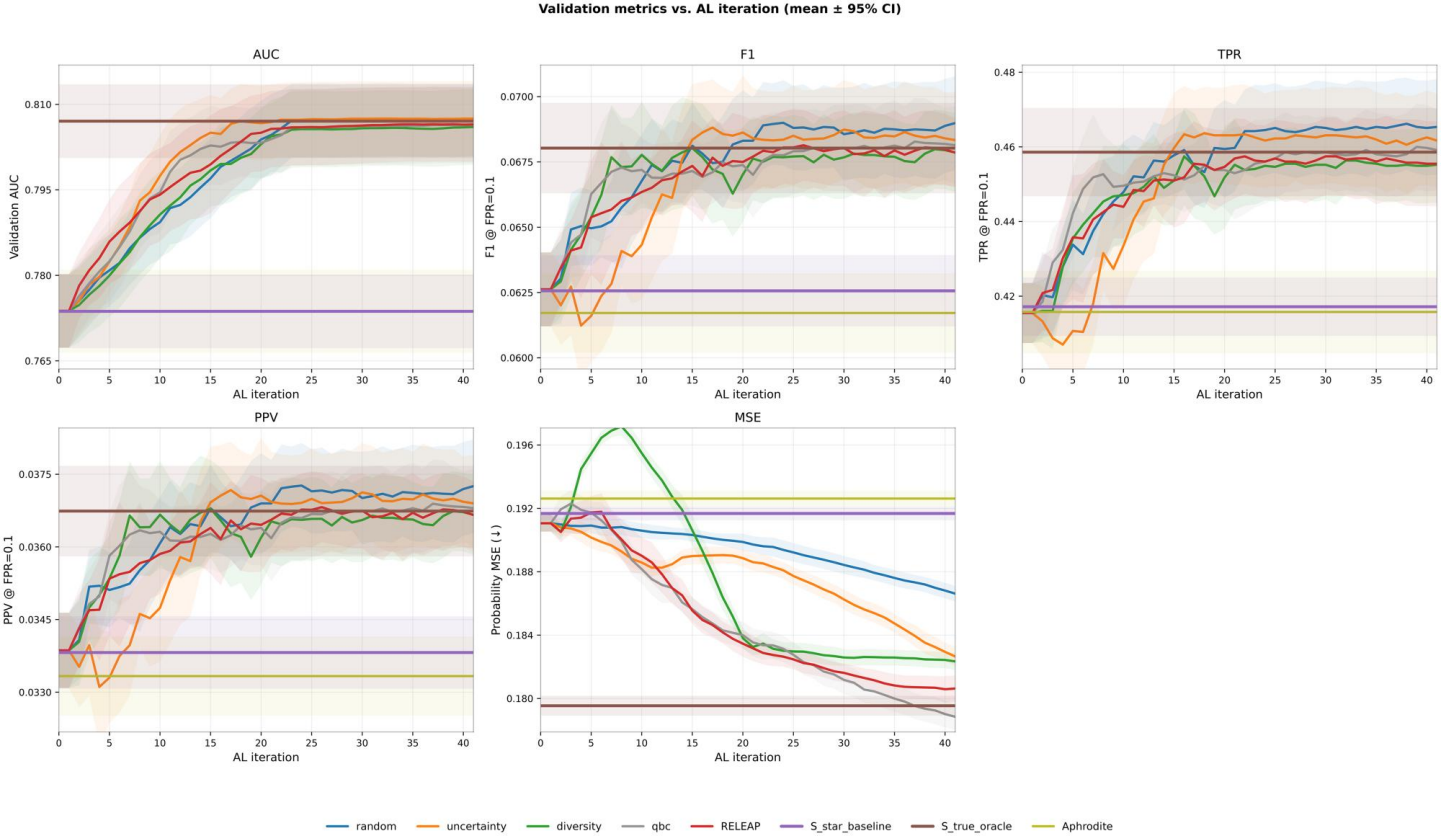
Validation metrics vs AL iteration in the logistic model (mean ± 95% CI over 10 replications). Abbreviation: AL, active learning.

Given the total cohort size of *N*=238 119, each experiment used a total labeling budget of 82 000 labeled training patients per replication. This corresponds to approximately 34.4% of the full cohort and about 43% of the training split (80% of *N*), leaving the remaining training patients with proxy labels. These labels were queried in 40 iterations of 2000 patients each, following an initial balanced seed set of 2000. All methods were repeated over 10 independent replications, where each replication re-split the cohort into stratified train/validation sets using a different random seed and reran the full pipeline (including model initialization and the active-learning query sequence). Figure 3 shows mean ± 95% CI trajectories for discrimination (AUC, F1 score, TPR, PPV) and calibration (probability MSE).

By the final iteration, discrimination improved markedly: mean AUC rose from 0.774±0.010 for the noisy proxy baseline to 0.807 with RELEAP and 0.807 with uncertainty sampling, closely approaching oracle-level performance (0.807). F1 and TPR showed comparable gains, while PPV increased modestly from 0.034 to 0.037. Calibration also improved, with MSE decreasing from 0.192 (baseline) to 0.181 under RELEAP, approaching the oracle’s 0.180.

Strategy behaviors diverged across metrics. *RELEAP* was generally competitive and often showed smoother, more stable trajectories across iterations. *Uncertainty* sampling achieved the largest gains during the early active-learning iterations (roughly the first 15 rounds) and then converged in AUC but showed more fluctuation in threshold-based metrics (F1/TPR/PPV) and calibration (MSE), as reflected by the more oscillatory trajectories in these panels. *Diversity* favored calibration but lagged slightly in discrimination, while *QBC* improved F1 score early before stabilizing. *Random* sampling improved gradually but remained inferior overall. RELEAP’s advantage lay in adaptively balancing these complementary heuristics.

#### Survival model (evaluated by C-index)

**Figure 4.**
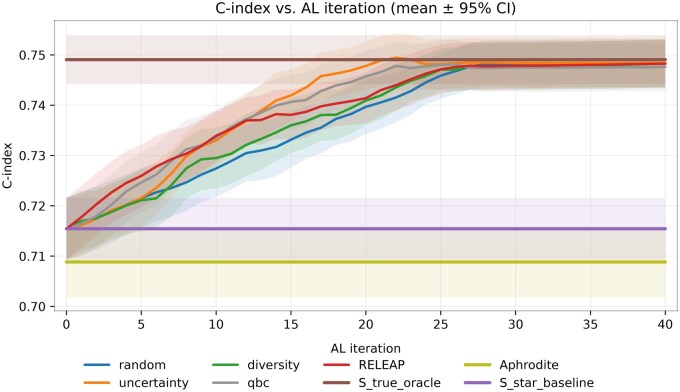
C-index vs AL iteration in the survival (Cox) model (mean ± 95% CI over 10 replications). Abbreviation: AL, active learning.

In the survival setting (penalized Cox regression; $Z=(S_{t},X_{2})$ after feature screening), experiments followed the same labeling budget of 82 000 patients (34.4% of the full cohort), queried in 40 iterations of 2000 over an initial balanced seed set of 2000. Each method was repeated 10 times. Figure 4 demonstrates that all strategies markedly outperformed the noisy proxy baseline (C-index 0.715, 95% CI, 0.709-0.721). RELEAP achieved 0.748 (0.743-0.753), converging toward the oracle (0.749, 0.744-0.754).

*Uncertainty* and *QBC* reached this plateau fastest, followed closely by *RELEAP*, while *diversity* and *random* trailed slightly. Overall, RELEAP provided the most balanced and reliable trajectory, achieving robust survival prediction with convergence, mirroring the efficiency observed in the logistic classification experiments.

### Subgroup analyses: male vs female

**Figure 5.**
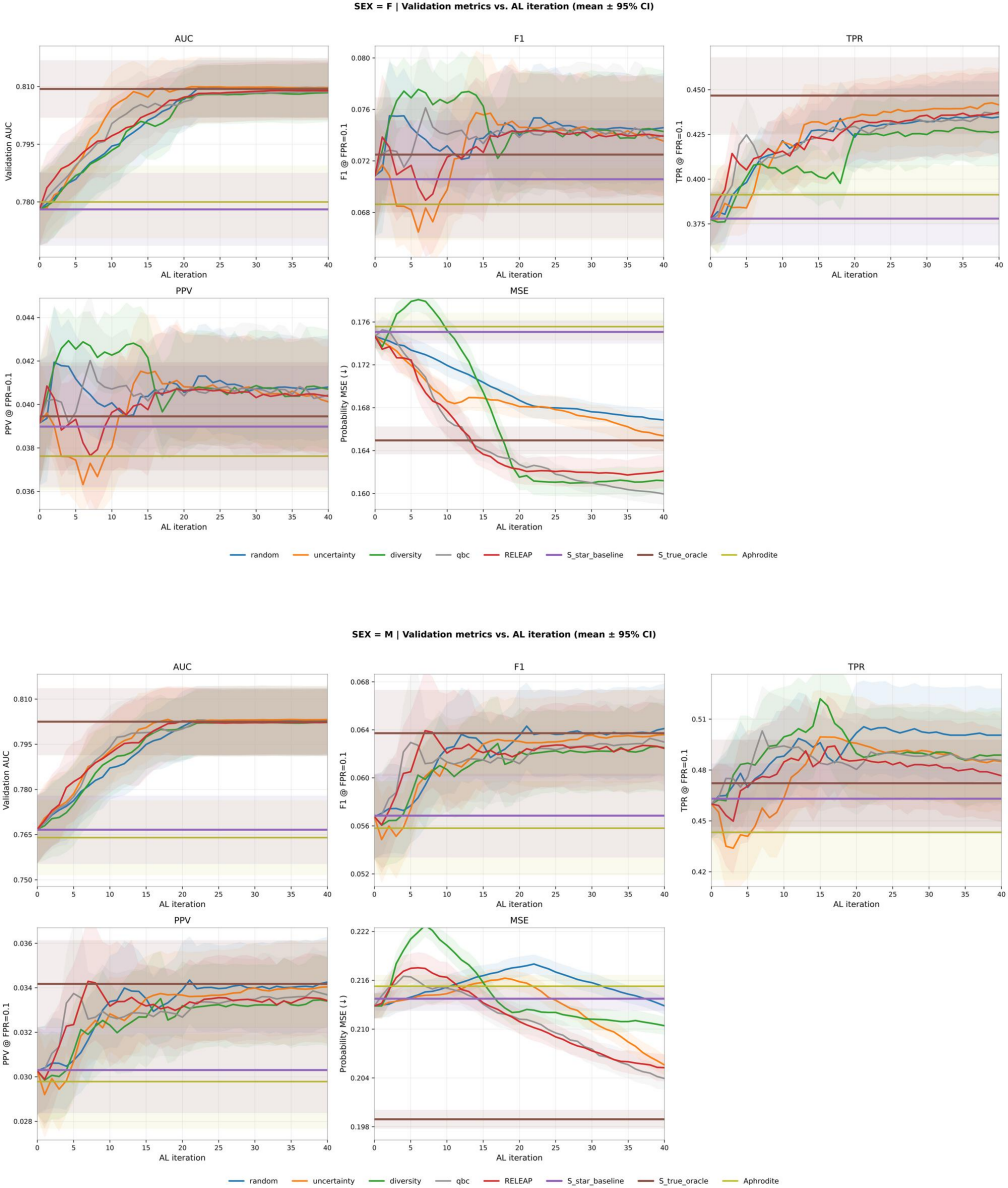
Validation metrics vs AL iteration in the logistic model (mean ± 95% CI over 10 replications) within male and female subgroups. Abbreviation: AL, active learning.

To further examine population heterogeneity, we stratified the analyses by sex. Figure 5 presents validation trajectories (mean ± 95% CI over 10 replications) for AUC, F1 score, TPR, PPV, and probability MSE in male and female subgroups.

#### Male subgroup

All active learning strategies improved steadily over the noisy proxy baseline (AUC 0.767±0.018) and approached the automatic reference phenotype oracle (0.803±0.018). Final AUC values clustered around 0.802-0.803 across methods, with RELEAP (0.802±0.018) and uncertainty sampling (0.803±0.018) leading. F1 increased modestly from 0.057±0.006 to about 0.062, with RELEAP and QBC showing smoother convergence. True positive rate rose from 0.463±0.034 to nearly 0.495 (uncertainty), while PPV improved slightly from 0.030 to 0.034. Calibration gains were limited: MSE decreased marginally from 0.213±0.002 to about 0.204 under RELEAP and QBC, remaining above the oracle (0.198±0.002). Overall, all strategies converged to similar discrimination and calibration levels, with RELEAP offering smoother and more stable trajectories rather than absolute superiority.

#### Female subgroup

In contrast, female subgroup results revealed clearer separation across strategies. Baseline AUC (0.778±0.015) improved to around 0.809-0.810 under RELEAP (0.809±0.012), uncertainty (0.810±0.012), and QBC (0.808±0.012), closely matching the oracle (0.810±0.012). F1 increased from 0.071±0.007 to 0.072-0.075, with RELEAP and QBC showing smoother trajectories, while diversity exhibited sharper fluctuations. True positive rate rose from 0.380±0.026 to above 0.436 for QBC, uncertainty, and RELEAP, approaching the oracle (0.446±0.036). Positive predictive value improved from 0.039±0.005 to approximately 0.041 under RELEAP, QBC, and uncertainty. Calibration gains were most pronounced in females: MSE decreased from 0.175±0.002 to near-oracle levels under RELEAP, QBC, and diversity (with slightly lower point estimates than the oracle), while random and uncertainty plateaued slightly higher (≈0.166).

#### Summary

In summary, while male subgroup results showed comparable convergence across strategies with limited calibration improvement, female subgroup results demonstrated clearer benefits from RELEAP. The RELEAP agent achieved balanced gains across discrimination, threshold, and calibration metrics, with the most stable convergence dynamics.

## Discussion

In this work, we proposed RELEAP, a reinforcement learning-enhanced agent for label-efficient active phenotyping. Unlike traditional active learning approaches that rely on a fixed querying strategy, RELEAP dynamically adjusts the weighting of multiple heuristics based on downstream model performance. By integrating uncertainty, diversity, and committee-based strategies within an RL-guided policy, RELEAP improves the quality of acquired labels and enhances predictive performance under constrained labeling budgets. The use of reinforcement learning further provides natural flexibility to incorporate multi-metric rewards, enabling balanced improvements in both discrimination and calibration.

Our real-world evaluation on the DUHS EHR cohort showed that RELEAP improved over the noisy proxy baseline and achieved performance close to the oracle under the same labeling budget. Across both logistic classification and survival analyses, RELEAP reduced the gap between proxy phenotypes and the automatic reference phenotype and yielded stable performance across discrimination, threshold-based metrics, and calibration. The sex-stratified subgroup analyses further illustrated that strategy differences can depend on the underlying data distribution: in settings where methods already converge quickly (eg, the male subgroup), many strategies reach a similar plateau, whereas in settings with greater heterogeneity (eg, the female subgroup), adaptive strategy mixing can yield smoother trajectories across metrics without committing to a single heuristic.

We emphasize that the sex-stratified analyses are descriptive and are intended to illustrate how querying behavior can differ under varying data distributions rather than to support clinical inference. A plausible explanation is limited headroom in the male subgroup: baseline performance was already close to the oracle and AUC curves largely converged across strategies, reducing the marginal benefit of entropy- or disagreement-driven querying. In contrast, the female subgroup exhibited larger early-iteration gaps and more heterogeneous trajectories across metrics, making sample selection more consequential. In such settings, RELEAP’s adaptive reweighting across uncertainty, QBC, and diversity can prioritize complementary signals over time and yield smoother convergence across discrimination, threshold-based metrics, and calibration rather than optimizing a single criterion alone.

Although RELEAP is designed to adaptively mix multiple querying heuristics, strict iteration-by-iteration dominance is not expected in this setting. Under batched querying and a finite labeling budget, performance gains can saturate, and several strong heuristics may converge to a similar plateau, particularly for AUC/C-index. In our phenotype-correction task, proxy-label errors are often aligned with model uncertainty and disagreement, so committee-based approaches such as QBC can identify many of the same high-impact patients as uncertainty-driven querying, leading to comparable downstream performance. We therefore interpret RELEAP’s primary benefit as robustness across metrics and settings: while fixed heuristics may converge similarly in AUC, they can exhibit more oscillatory behavior in threshold-based metrics (F1/TPR/PPV) and calibration (MSE), whereas RELEAP can rebalance strategy weights to stabilize trajectories when the optimal querying behavior shifts.

We observed that random sampling improves gradually over longer horizons and can move toward oracle-level discrimination as more patients are eventually upgraded from proxy labels to reference labels. This behavior is consistent with the fact that random sampling will eventually cover a broad portion of the feature space when the budget is sufficiently large, narrowing the gap in discrimination metrics that are less sensitive to which specific patients are selected early. However, random sampling remains less competitive in efficiency and in metrics that depend more strongly on the composition of the labeled set at each iteration, such as calibration and threshold-based measures, where targeted selection can provide more consistent improvements under the same budget.

This study has several limitations. First, RELEAP was validated in the context of smoking phenotype correction for lung cancer risk prediction; additional evaluations are needed to assess generalizability across other phenotyping targets, outcomes, and noise structures. Second, the current implementation used data from a single health system, and external validation across sites would be important given coding and documentation variability. Third, some structured covariates rely on diagnosis code ascertainment (eg, smoking-related ICD codes used for the proxy phenotype and ICD-based COPD indicators), which may be subject to misclassification and site-specific coding practices; while we anchor smoking evaluation to a multimodal automatic reference phenotype, similar measurement error may remain for other coded comorbidities. Fourth, the finite labeling budget limits the amount of feedback available for policy learning, which can reduce the margin over strong fixed heuristics in regimes where performance quickly saturates. Finally, we considered 3 active learning strategies; additional heuristics and feature modalities may further enrich the policy space.

Future work should extend RELEAP to a broader set of phenotype correction tasks, including behavioral, genomic, and imaging-derived phenotypes. Beyond phenotyping, the agent could be applied to downstream applications such as risk stratification, treatment effect heterogeneity, and trial eligibility screening, where label efficiency and adaptivity are equally important. Incorporating richer reward functions that balance fairness, interpretability, and cost-effectiveness may also further enhance RELEAP’s impact in real-world clinical decision support.

## Conclusion

RELEAP provides a practical framework for adaptive, label-efficient phenotyping. By using downstream model performance as a feedback signal, RELEAP dynamically refines phenotype labels to enhance risk prediction accuracy and model reliability in real-world EHR settings.

## Supplementary Material

ooag019_Supplementary_Data

## Data Availability

All code (including scripts to reproduce the figures) is publicly available at https://github.com/yy447/AL_framework. Because the DUHS EHR data contain protected health information, they cannot be shared. Aggregate summary statistics and code lists used for cohort construction are provided in Supplementary Sections S0-S6.
