# Supplementary material for: RELEAP: reinforcement-enhanced label-efficient active phenotyping for electronic health records
Source: JAMIA Open. 2026 Feb 18;9(1):ooag019. doi: 10.1093/jamiaopen/ooag019 (PMC12918302; doi:10.1093/jamiaopen/ooag019)
Supplement: ooag019_Supplementary_Data [file ooag019_supplementary_data.zip › RELEAP_supplement_clean.pdf]

S0. Notation and Conventions

To reduce cognitive load, we summarize all symbols used throughout the Supplementary Material.

Indexing and sets.

- $i = 1, \dots, n$: patient index; $t = 1, \dots, H$: active learning / RL iteration index.
- $\mathcal{L}_t$: labeled set after iteration t (queried patients with reference labels revealed).
- $\mathcal{U}_t$: unlabeled pool after iteration t (remaining candidates).
- k: batch size queried at each iteration.

Features and labels.

- $X_{1,i}$: structured inputs used to build the proxy phenotype (e.g., smoking ICD codes).
- $X_{2,i}$: structured covariates used in the downstream risk model (e.g., demographics, COPD).
- $S_{\text{true},i}$: automatic reference phenotype (higher-fidelity label; revealed only when queried).
- S_i^*: proxy phenotype (noisy, widely available; continuous score in our experiments).
- S_t: the phenotype label vector used to train the downstream model at iteration t. Its ith entry is

$$S_{t,i} = \begin{cases} S_{\text{true},i}, & i \in \mathcal{L}_t, \\ S_i^*, & i \in \mathcal{U}_t. \end{cases}$$

Outcomes and evaluation.

- $Y_i \in \{0, 1\}$: binary outcome (e.g., incident lung cancer).
- T_i: time-to-event outcome for patient i (used in survival analysis).
- m_t: downstream validation metric at iteration t (AUC for logistic, C-index for Cox).

RL variables.

- s_t: constructed summary state (feature vector) at iteration t.
- $\boldsymbol{\ell}_t^{\text{strat}}$: strategy-summary features at iteration t, defined as the concatenation of per-strategy distribution summaries on $\mathcal{U}_t$ (median and 80th percentile for uncertainty/diversity/QBC scores).
- a_t: action at iteration t; in our setting, $a_t \equiv \mathbf{w}_t \in \Delta^2$.
- $\mathbf{w}_t = (w_t^{\text{unc}}, w_t^{\text{div}}, w_t^{\text{qbc}})$: nonnegative simplex weights over AL strategies.
- R_t: scalar reward at iteration t derived from m_t.
- $\pi_\theta(a \mid s)$: stochastic policy parameterized by θ.
- $\gamma \in (0, 1]$: discount factor; H: episode horizon (budget exhaustion).

Moving averages and windows.

- W: moving-average window length used for reward stabilization.
- ε: small constant for numerical stability.

S1. Simulation Study

Simulation Setting

We evaluated the finite-sample performance of the proposed RL–AL framework using synthetic data generated under the minimal causal structure $X_1 \to S_{\text{true}} \to S^*$ and $(S_{\text{true}}, X_2) \to Y$. Across iterations, the downstream model is trained using the evolving phenotype label vector S_t defined in Section S0.

- **Sample size.** For each replication, we generate $n = 1000$ patients and split them into 80% training and 20% validation sets stratified by Y.
- **Predictors.** Draw $X_{1,i} \in \mathbb{R}^{d_{X1}}$ and $X_{2,i} \in \mathbb{R}^{d_{X2}}$ independently from standard Gaussian distributions.
- **Reference phenotype.** Generate $S_{\text{true},i} \sim \text{Bernoulli}(\sigma(\eta_i))$ where $\eta_i = \beta_0 + X_{1,i}^\top \beta_1 + \xi_i$, $\xi_i \sim \mathcal{N}(0, \sigma_\xi^2)$ and $\sigma(\cdot)$ is the sigmoid.
- **Proxy phenotype.** Generate a noisy continuous proxy $S_i^* = \sigma(\alpha_0 + \alpha_1 S_{\text{true},i} + \epsilon_i)$ with $\epsilon_i \sim \mathcal{N}(0, \sigma_\epsilon^2)$.
- **Outcome.** Generate $Y_i \sim \text{Bernoulli}(\sigma(\theta_0 + \theta_S S_{\text{true},i} + X_{2,i}^\top \theta_2))$.

Outcome prevalence scenarios. To assess robustness to outcome prevalence, we varied the marginal prevalence of Y across three representative settings: {1%, 10%, 30%}, corresponding to rare, moderate, and common outcomes. Operationally, for each target prevalence p_Y, we calibrated the outcome intercept θ_0 (e.g., via bisection search) so that the empirical mean of $\sigma(\theta_0 + \theta_S S_{\text{true},i} + X_{2,i}^\top \theta_2)$ approximately matches p_Y, while keeping all other data-generating parameters fixed.

We compared random sampling, uncertainty, diversity, query-by-committee (QBC), and RELEAP under identical labeling budgets and batch sizes.

Simulation Results

Figures 1–3 summarize validation Metrics across active-learning iterations (mean ± 95% CI over 100 replications) under three outcome prevalence settings. Across all settings, active learning improved over the noisy proxy baseline (training on S^* only) and moved toward the oracle benchmark (training on S_{true} for all samples).

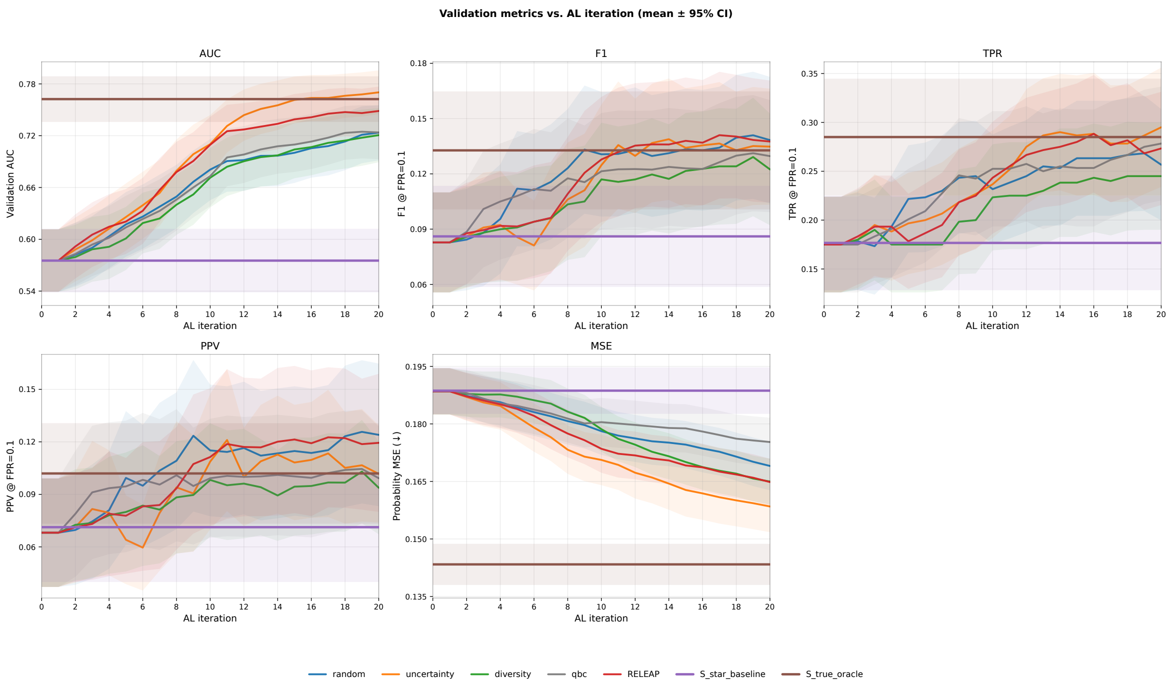

Figure 1: Simulation (target prevalence = 1%): validation Metrics vs. AL iteration (mean ± 95% CI over 100 replications).

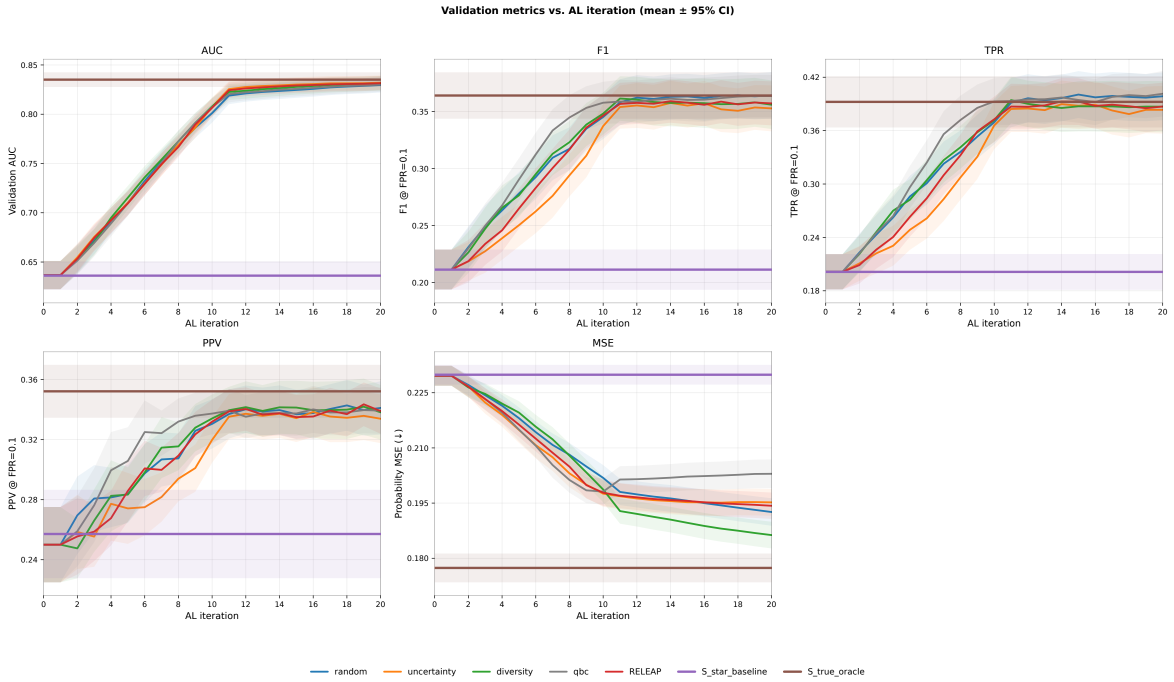

Figure 2: Simulation (target prevalence = 10%): validation Metrics vs. AL iteration (mean ± 95% CI over 100 replications).

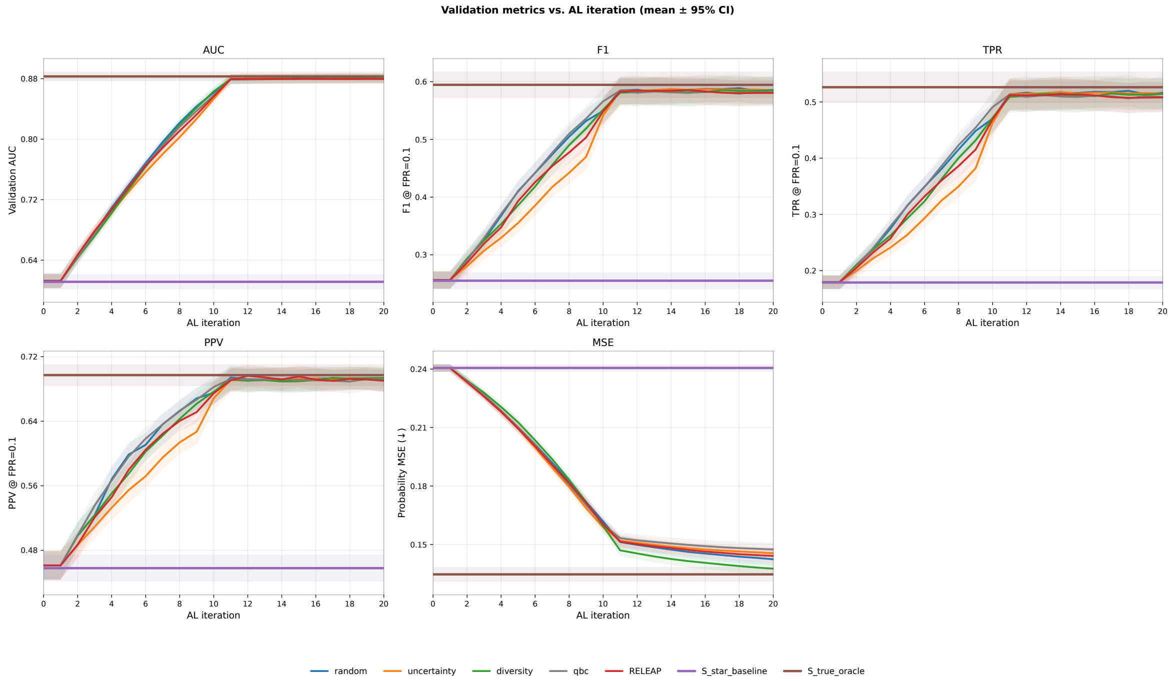

Figure 3: Simulation (target prevalence = 30%): validation Metrics vs. AL iteration (mean ± 95% CI over 100 replications).

Effect of outcome prevalence. Outcome prevalence materially influenced the separation among strategies. When Y was rare (1%), strategy differences were more pronounced and performance gains accrued more gradually, making sample selection more consequential. As prevalence increased (10% and 30%), the task became easier and all strategies rapidly approached the oracle performance, leading to substantially smaller between-strategy gaps. This sensitivity suggests that the relative advantage of adaptive strategy mixing is most evident in more challenging, low-prevalence settings that resemble the lung cancer application.

S2. Active Learning Strategies

At each iteration t, we train the downstream model on the full training set using the current phenotype vector S_t, where $S_{t,i} = S_{\text{true},i}$ for $i \in \mathcal{L}_t$ and $S_{t,i} = S_i^*$ for $i \in \mathcal{U}_t$. We then score each $i \in \mathcal{U}_t$ for acquisition. Each patient is represented by the feature vector $[S_{t,i}, X_{2,i}]$, where $S_{t,i}$ follows the definition in Section S0.

Uncertainty

Let f_t denote the fitted logistic model at iteration t. For each unlabeled patient $i \in \mathcal{U}_t$, let $\hat{p}_{t,i} = f_t([S_{t,i}, X_{2,i}])$. We define the uncertainty score as the Shannon entropy:

$$H(\hat{p}_{t,i}) = -\hat{p}_{t,i} \log \hat{p}_{t,i} - (1 - \hat{p}_{t,i}) \log(1 - \hat{p}_{t,i}),$$

where larger values indicate less confident predictions.

Diversity

We compute cosine distances in the standardized $[S_t, X_2]$ space between each unlabeled patient $i \in \mathcal{U}_t$ and its $K_{\text{nn}} = 10$ nearest labeled neighbors in $\mathcal{L}_t$. Let d_{ij} denote the cosine distance between i and neighbor j. The diversity score is

$$D_{t,i} = \text{mean}(d_{ij}) + \lambda \, \text{std}(d_{ij}), \quad \lambda = 0.5.$$

Query-by-Committee (QBC)

We train a committee of $M = 7$ logistic models $\{f_t^{(m)}\}_{m=1}^{M}$ on bootstrap resamples of $\mathcal{L}_t$. For each $i \in \mathcal{U}_t$, let $\hat{p}_{t,i}^{(m)} = f_t^{(m)}([S_{t,i}, X_{2,i}])$. We define the QBC disagreement score as

$$Q_{t,i} = \text{Var}_m \big[\hat{p}_{t,i}^{(m)}\big],$$

with a small entropy stabilizer added in implementation to avoid degenerate variance when predictions are extreme.

Random baseline

Random sampling uniformly selects k patients from $\mathcal{U}_t$ and serves as a lower-bound reference.

S3. Reinforcement Learning Formulas

At iteration t, the RL agent observes a summary state s_t, selects an action $a_t \equiv \mathbf{w}_t \in \Delta^2$, and receives a reward R_t based on downstream validation performance m_t.

State

We construct s_t as a concatenated feature vector:

$$s_t = \Big[m_t,\ \boldsymbol{\ell}_t^{\text{strat}},\ \mu_t^{(S)},\ \sigma_t^{(S)},\ \text{slope}(m),\ \text{var}(m),\ b_t \Big],$$

where

- m_t is the current downstream validation metric (AUC for logistic regression or C-index for Cox);
- $\boldsymbol{\ell}_t^{\text{strat}}$ summarizes per-strategy score distributions on $\mathcal{U}_t$ (median and 80th percentile for uncertainty, diversity, and QBC scores);
- $\mu_t^{(S)}$ and $\sigma_t^{(S)}$ are the mean and standard deviation of $\{S_{t,i} : i \in \mathcal{L}_t\}$;
- slope(m) and var(m) summarize short-term trends and variability of recent metric values;
- b_t is the fraction of labeling budget remaining.

Action

The action is a simplex weight vector

$$\mathbf{w}_t = (w_t^{\text{unc}}, w_t^{\text{div}}, w_t^{\text{qbc}}), \quad w_t^{\text{unc}}, w_t^{\text{div}}, w_t^{\text{qbc}} \geq 0, \quad w_t^{\text{unc}} + w_t^{\text{div}} + w_t^{\text{qbc}} = 1,$$

which linearly combines normalized strategy scores to rank patients in $\mathcal{U}_t$. In implementation, the policy network outputs logits transformed by softmax to ensure $\mathbf{w}_t \in \Delta^2$.

Reward

Let $\bar{m}_t$ denote the moving average of $\{m_{t-W+1}, \ldots, m_t\}$ over window length W. We define the relative gain

$$g_t = \frac{m_t - \bar{m}_t}{1 - \bar{m}_t + \varepsilon},$$

and the shaped raw reward

$$R_t^{\text{raw}} = g_t \, (1 + 2 \, \text{prog}_t) \, \tau_t,$$

where prog_t is the fraction of budget already used and $\tau_t \in \{1.0, 1.2\}$ upweights improving trends. We then normalize online:

$$R_t = \frac{R_t^{\text{raw}} - \mu_t}{\sigma_t + \varepsilon},$$

with running mean μ_t and running standard deviation σ_t of R_t^{raw}.

RL Algorithm

We optimize the policy using Proximal Policy Optimization (PPO) to maximize the expected discounted return

$$\max_\theta \; \mathbb{E}\Big[\sum_{t=1}^{H} \gamma^{t-1} R_t\Big],$$

where $\gamma \in (0, 1]$ and episodes terminate at horizon H when the labeling budget is exhausted.

S4. Mathematical Formulation (Compact)

Let $\mathcal{A} = \{\text{uncertainty}, \text{diversity}, \text{QBC}\}$ denote the candidate acquisition strategies. At iteration t, the agent observes

$$s_t = \Big[m_t,\ \boldsymbol{\ell}_t^{\text{strat}},\ \mu_t^{(S)},\ \sigma_t^{(S)},\ \text{slope}(m),\ \text{var}(m),\ b_t\Big],$$

selects $\mathbf{w}_t \in \Delta^2$, and induces a ranking score for each $i \in \mathcal{U}_t$ by a weighted combination of normalized strategy scores. The reward R_t is defined in Section S3, and PPO updates $\pi_\theta(a \mid s)$ to maximize $\mathbb{E}[\sum_{t=1}^{H} \gamma^{t-1} R_t]$.

S5. Benchmark Smoking Phenotype Variants (Self-report and LLM/NLP Comparisons)

To address reviewer-suggested benchmarks, we evaluated additional smoking phenotype variants that incorporate (1) structured self-reported smoking information and (2) smoking status extracted from clinical notes using either traditional NLP concept identifiers (CUIs) or a large language model (LLM). These variants are used as reference/benchmark phenotype definitions to contextualize the performance of RELEAP, and are reported alongside the ICD-based proxy phenotype (S^*).

Structured self-reported smoking (SR). Structured smoking status was obtained from DUHS vital signs / social history fields when available. For consistency with the main experiments, SR was mapped to a binary indicator of smoking history (ever vs. never). (When SR was missing, the variant definition relied on the remaining available signals as described below.)

Text-derived smoking signals from notes. We considered two complementary approaches: (i) **NLP CUIs**, i.e., smoking-related Concept Unique Identifiers extracted from clinical notes using a traditional dictionary-/rule-based pipeline; and (ii) **LLM mentions**, i.e., smoking-related mentions extracted from notes using an LLM-based extraction procedure.

Benchmark phenotype variants. We evaluated the following variants (matching the labels in Figure 4):

- S^* **baseline (ICD proxy):** Smoking proxy constructed exclusively from smoking-related ICD codes (Table S**??**).
- $S_{\mathbf{true}}$ **v1 (SR + CUIs):** Structured self-report combined with NLP-derived CUIs from notes.
- $S_{\mathbf{true}}$ **v2 (SR + LLM):** Structured self-report combined with LLM-extracted note mentions.
- $S_{\mathbf{true}}$ **v3 (No SR + CUIs):** NLP-derived CUIs only (no structured self-report).
- $S_{\mathbf{true}}$ **v4 (No SR + LLM):** LLM-extracted mentions only (no structured self-report).
- $S_{\mathbf{true}}$ **v5 (Only SR):** Structured self-report only, without any text-derived signals.

Figure 4 summarizes validation performance across discrimination (AUC), threshold-based metrics (F1, TPR, PPV), and calibration (MSE of predicted probabilities). Overall, variants that integrated structured self-report with either NLP CUIs or LLM-derived mentions (S_{true} v1–v2) achieved the strongest performance, while text-only definitions (v3–v4) were consistently weaker. The SR-only definition (v5) improved

over the ICD proxy but was generally inferior to SR+text variants, highlighting the complementary value of note-derived smoking signals.

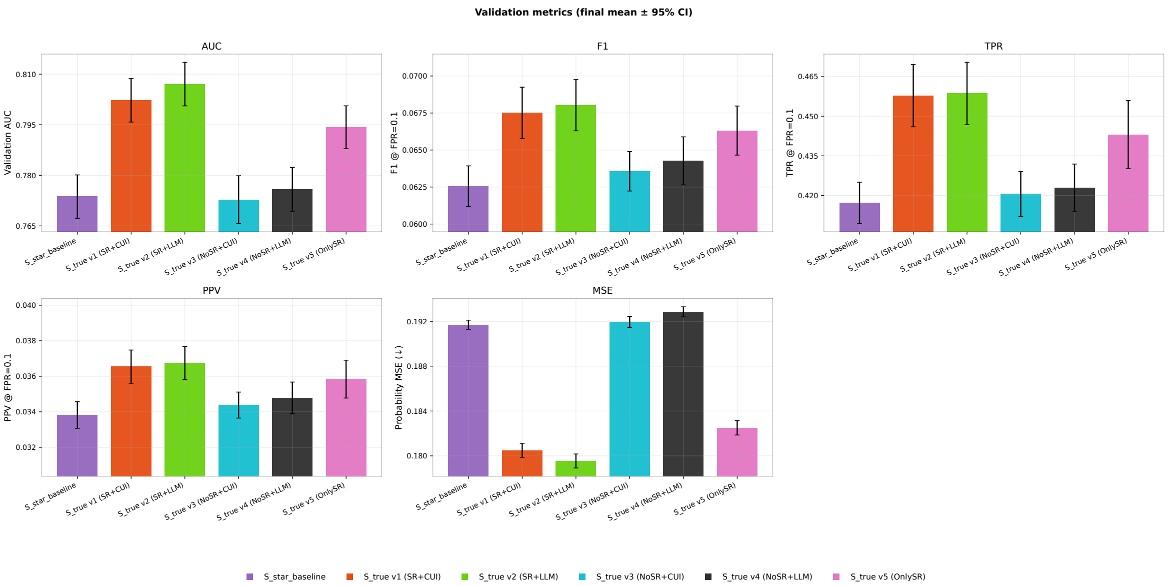

Figure 4: Benchmark smoking phenotype variants for reviewer-requested comparisons. Bars show mean validation performance (AUC, F1, TPR, PPV, MSE) with 95% CI error bars for the ICD proxy baseline (S^*) and variants incorporating structured self-report (SR) and note-derived smoking signals from traditional NLP CUIs or LLM extraction.

S6. DUHS Lung Cancer Cohort Construction and Code Lists

This section provides the detailed cohort construction rules and complete code lists used in the real-world DUHS lung cancer prediction experiments. These details are provided to ensure full reproducibility and to clarify all references to "qualifying codes," exclusion criteria, and phenotype definitions used in the main text.

S6.1 Cohort Entry and Eligibility Criteria

The lung cancer cohort was constructed following established EHR-based cohort definitions [?], with minor adaptations to the DUHS data structure.

Index date. The index date was defined as the first qualifying outpatient encounter occurring between 2016 and 2018.

Inclusion criteria. Patients were required to meet all of the following:

- Age between 45 and 65 years at index date.
- At least one outpatient encounter during the index window (2016–2018).
- At least 365 days of EHR history prior to the index date.

Exclusion criteria. Patients were excluded if they met any of the following:

- Any history of cancer prior to index, except nonmelanoma skin cancer (code lists in Table S7).
- Evidence of lung cancer–related diagnostic evaluation, procedures, or treatment prior to index (code lists in Table S8).
- No follow-up encounters or observations after the index date, indicating insufficient longitudinal data.

S6.2 Outcome Ascertainment

The primary outcome was incident lung cancer occurring after the index date and before the administrative study end date. Lung cancer was defined by the presence of at least two primary lung cancer diagnosis codes occurring within 60 days of each other (Table S6). Patients with any qualifying lung cancer diagnosis prior to index were excluded. Patients without an event were censored at the study end date.

S6.3 Lung Cancer Outcome Codes

Table 1: Primary lung cancer ICD codes used for outcome definition.

Code system	Codes
ICD-9	162.xx (malignant neoplasm of trachea, bronchus, and lung)
ICD-10	C34.xx (malignant neoplasm of bronchus and lung)

S6.4 Cancer History Exclusion Codes

Table 2: Cancer history exclusion codes (excluding nonmelanoma skin cancer).

Code system	Codes
ICD-9	140–239 (neoplasms), excluding 173.xx
ICD-10	C00–D49 (neoplasms), excluding C44.xx

S6.5 Lung Cancer–Related Evaluation, Procedures, and Treatment Prior to Index

Patients with evidence of lung cancer–related evaluation, procedures, or treatment prior to index were excluded to reduce reverse causation and ensure incident outcome ascertainment.

Table 3: Codes indicating lung cancer–related evaluation, procedures, or treatment prior to index.

**Category**	**Codes**
Chest CT (screening/diagnostic)	CPT: 71250, 71260, 71270; HCPCS: G0297
PET imaging	CPT: 78811, 78812, 78813, 78814
Biopsy (lung/bronchus)	CPT: 32405, 32408, 32607, 32608, 32609, 31628, 31625
Bronchoscopy	CPT: 31622–31629
Surgical resection	ICD-9 Procedure: 32.xx; CPT: 32480, 32482, 32488
Radiation therapy	CPT: 77401–77499; HCPCS: G0339, G0340, G6003–G6014
Ablation	CPT: 32994
Systemic therapy (chemotherapy)	ICD-9: V58.1, V66.2; ICD-10: Z51.11, Z51.12; HCPCS: J9000–J9999
Hemoptysis (symptom)	ICD-9: 786.3; ICD-10: R04.2

S6.6 Smoking Proxy Phenotype (S^*) ICD Codes

The noisy proxy smoking phenotype (S^*) was constructed exclusively from smoking-related ICD codes to reflect a widely available but incomplete structured signal in real-world EHR data.

Table 4: Smoking-related ICD codes used to construct the proxy phenotype S^*.

**Code system**	**Codes**
ICD-9	305.1 (tobacco use disorder), V15.82 (history of tobacco use)
ICD-10	F17.*, Z72.0, Z87.891, Z71.6

S6.7 COPD Definition Codes

Chronic obstructive pulmonary disease (COPD) was defined using the following ICD codes. Similar to smoking-related ICD codes, COPD coding may be subject to misclassification and was treated as a structured covariate rather than a gold-standard phenotype.

Table 5: ICD codes used to define COPD.

**Code system**	**Codes**
ICD-9	490, 491.xx, 492.xx, 494.xx, 496
ICD-10	J40, J41.xx, J42, J43.xx, J44.xx, J47.xx
